# Occupational cataracts and lens opacities in interventional cardiology (O'CLOC study): are X-Rays involved?

**DOI:** 10.1186/1471-2458-10-537

**Published:** 2010-09-08

**Authors:** Sophie Jacob, Morgane Michel, Christian Spaulding, Serge Boveda, Olivier Bar, Antoine P Brézin, Maté Streho, Carlo Maccia, Pascale Scanff, Dominique Laurier, Marie-Odile Bernier

**Affiliations:** 1 IRSN - DRPH/SRBE/Laboratoire d'Epidémiologie, Fontenay-aux-Roses, France; 2 Cochin Hospital, Paris Descartes University and INSERM U 909, Paris France; 3 Clinique Pasteur, Toulouse, and Groupe Rythmologie Stimulation Cardiaque/SFC, France; 4 Clinique St Gatien, Tours, and Groupe Athérome Cardiologie Interventionnelle/SFC, France; 5 Paris Descartes University APHP Centre Cochin Ambulatoire d'Ophtalmologie, Paris, France; 6 Centre d'Assurance de qualité des Applications Technologiques dans le domaine de la Santé, Bourg-La-Reine, France; 7 IRSN - DRPH/SER/Unité de Suivi et d'analyses des Expositions Professionnelles, Fontenay-aux-Roses, France

## Abstract

**Background:**

The eye is well known to be sensitive to clearly high doses (>2 Gy) of ionizing radiation. In recent years, however, cataracts have been observed in populations exposed to lower doses. Interventional cardiologists are repeatedly and acutely exposed to scattered ionizing radiation (X-rays) during the diagnostic and therapeutic procedures they perform. These "low" exposures may cause damage to the lens of the eye and induce early cataracts, known as radiation-induced cataracts. The O'CLOC study (Occupational Cataracts and Lens Opacities in interventional Cardiology) was designed to test the hypothesis that interventional cardiologists, compared with an unexposed reference group of non-interventional cardiologists, have an increased risk of cataracts.

**Method/Design:**

The O'CLOC study is a cross-sectional study that will include a total of 300 cardiologists aged at least 40 years: one group of exposed interventional cardiologists and another of non-interventional cardiologists. The groups will be matched for age and sex. Individual information, including risk factors for cataracts (age, diabetes, myopia, etc.), will be collected during a telephone interview. A specific section of the questionnaire for the exposed group focuses on occupational history, including a description of the procedures (type, frequency, radiation protection tool) used. These data will be used to classify subjects into "exposure level" groups according to cumulative dose estimates. Eye examinations for all participants will be performed to detect cataracts, even in the early stages (lens opacities, according to LOCS III, the international standard classification).  The analysis will provide an estimation of the cataract risk in interventional cardiology compared with the unexposed reference group, while taking other risk factors into account. An analysis comparing the risks according to level of exposure is also planned.

**Discussion:**

This epidemiological study will provide further evidence about the potential risk of radiation-induced cataracts at low doses and contribute to cardiologists' awareness of the importance of radiation protection.

**Trial Registration:**

NCT01061463

## Background

Although the sensitivity of the lens of the eye to high doses of ionizing radiation (IR) is well known, considerable uncertainty surrounds the relation between radiation dose and cataracts. The radiation protection standards formulated by the United States National Council on Radiation Protection (NCRP) and the International Commission on Radiological Protection (ICRP) [1] are all based on the assumption that lens opacities (cataracts) are deterministic radiation-induced effects and appear only if a dose threshold is exceeded [1,2]. The current dose thresholds for radiation-induced lens opacities are 2 Gy for a single dose or 5 Gy for fractionated doses. However, several recent studies have now raised questions about this assumption [3] (UNSCEAR (United Nations Scientific Committee on the Effects of Atomic Radiation) 2008 Report: *"Sources of ionizing radiation"*.). Epidemiological and experimental studies appear to show the formation of radiation-induced cataracts at much lower doses than the current standards and strongly suggest a stochastic hypothesis (non-threshold effect) [4].

### Radiation-induced cataracts in populations with low levels of radiation exposure

While posterior subcapsular cataracts are characteristic of radiation exposure, several sets of data suggest that the broader category of posterior cortical cataracts may also be regarded as radiation-associated. Increased risks of lens opacities (including posterior subcapsular, cortical, nuclear, and mixed cataracts) have been reported in different populations for the lower doses induced by chronic, fractionated, or acute exposure to γ or neutron radiation, β particles, galactic cosmic radiation, and X-rays (Table 1). An increased frequency of radiation-induced lens opacities has also been observed in a variety of environmental, medical, and occupational contexts. Reports of lens opacities related to environmental exposure come from the Hiroshima and Nagasaki survivors [5-7], from children living in the contaminated territories of Chernobyl [8], and residents of (60)Co-contaminated buildings in Taiwan [9]. Sources of medical exposure include X-Ray exposure during computed tomography [10] and radiotherapy [11,12]. Occupational exposure to IR and lens opacities have been reported for Chernobyl clean-up workers [13], astronauts [14-16] and pilots [17], and medical personnel, such as radiology technicians [18]. Nonetheless, new data from exposed human populations are still necessary to confirm the absence of a dose threshold, or the need to revise the existing threshold.

**Table 1:** Main epidemiological studies of low dose radiation-induced cataracts

	Population size	Exposure level	Exposure age or period	Eye examination age or period	Type of cataracts involved
**Hiroshima and Nagasaki survivors**					
*Otake et al. *[5]	Cohort of 1983 individuals	-	H & N: Mean age = 29.3 yrs and 23.4 yrs	1963-1964	γ-ray threshold: 730 mGy neutron-ray threshold: 60 mGy
*Minamoto et al.*[6]	Cohort of 873 individuals	Eye dose= 405 mSv	Mean age = 8.8 yrs	Mean age = 64.8 yrs	Cortical opacities PS opacities
*Nakashima et al.*[7]	Cohort of 730 individuals	-	Median age = 10.5 yrs	Median age = 66.6 yrs	Threshold dose: 0.6 Sv for cortical opacities 0.7 Sv for PS cataracts

**Chernobyl children**					
*Day et al.*[8]	996 exposed/791 unexposed	-	Chronic	Range = 5 - 17 yrs	PS opacities

**Contaminated buildings in Taiwan**					
*Chen et al.*[9]	Cohort of 114 individuals	161.9 mSv	Chronic	Mean= 24.8 yrs	Focal lens defects

**Diagnostic examinations**					
*Klein et al.*[10]	4926 subjects	Diagnostic X-Ray exposure	-	Range = 43 - 84 yrs	PS opacities

**Treatment of benign diseases**					
*Wilde et al.*[12]	20 adults treated by radium irradiation	Range: 1-8 Gy	Median age = 6 months	Range = 31 - 46 yrs	Subcapsular punctuate opacities at 100 mGy
*Hall et al. *[11]	483 individuals treated by radiotherapy vs. 89 controls	0.4 Gy (0-8.4)	Mean age = 5 months	Range = 36 - 54 yrs	PS opacities and Cortical opacities

**Chernobyl Liquidators**					
*Worgul et al.*[13]	8,607 Ukrainian workers	Median lens dose = 120 mGy (0-0.8)	Mean age = 36.7 yrs	Mean = 45 yrs	PS changes and Cortical cataracts

**Astronauts**					
*Cucinotta et al*[14]	295 astronauts	Mean eye dose = 3.6 mSv	Chronic (at least 40 yrs old at first flight)	First eye exam in 1977	PS cataracts, Nuclear cataracts and mixed
*Rastegar et al.*[15]	21 astronauts vs. 395 unexposed	-	Mean time in space = 62 days	Mean = 59 yrs in astronauts group	Opacities in posterior capsule
*Chylack et al.*[16]	171 astronauts vs. 247 unexposed	Lens dose = 15.1 to 129.3 mSv	-	2004-2006	Cortical cataracts and PS opacities

**Airline pilots**					
*Rafnsonn et al.*[17]	274 pilots with lens opacities vs. 374 controls	Cumulative dose: 0 to 48 mSv	-	Mean = 75 yrs in cases; 66.1 yrs in controls	Nuclear cataract

**Radiologists and radiological technologists**					
*Chodick et al.*[18]	Cohort of 35 705 radiology technicians	Median = 28.1 mGy	Range = 24 - 44 yrs	Follow up between 1983 and 2004	Any cataract

PSC: posterior subcapsular cataract; PS: posterior subcapsular;

### Interventional cardiologists: a little-studied exposed population

The widespread use of IR in medical practice for both diagnostic and therapeutic purposes results in a significant increase in exposure of both patients and medical staff [19]. The use of medical imaging involving X-rays as a diagnostic tool or during interventional procedures has increased steadily over the last few years, particularly in the field of interventional cardiology, including cardiac electrophysiology [20,21]. Interventional cardiologists are now thought to be the most highly exposed of all medical personel [22-24]. The ablation of atrial fibrillation performed by electrophysiologists, for example, is a long and potentially irradiating procedure [25], and the operator's eyes are exposed to scattered X-rays. The frequent failure of some cardiologists to use protective leaded eyewear helps explain the crucial need for radiation monitoring and risk assessment for medical staff [26]. Interventional cardiologists are exposed to risks in the same range as those for which early-stage cataracts have been observed.

Very few epidemiological studies have been published on the risk of cataracts in interventional radiology (including procedures for interventional cardiology). Junk *et al. *[27] took a first step towards identifying and increasing awareness of these risks in a study that screened 59 volunteer participants at a professional meeting of interventional radiologists, including cardiologists. They observed a surprisingly high frequency of posterior subcapsular cataracts in their sample: 22 individuals (37.3%) had small paracentral dot-like opacities in the posterior subcapsular region of the lens, consistent with early signs of radiation damage, and five more (8%) had diagnoses of cataracts (corresponding to more advanced stages of lens opacities). This study nevertheless had limitations: a selection bias due to their recruitment method, which may have resulted in overestimating prevalence, and the absence of a control group of unexposed participants. A study presented at the European Society of Cardiology congress in 2009 [28] did use both an exposed and a control group and found a significant difference in the frequency of lens opacities (37.9% vs. 12%, p < 0.005). These findings reinforced the results reported by Junk *et al. *Nevertheless, as the authors underlined, their study also had methodological limitations, including the same potential selection bias described for Junk *et al. *and an age difference between exposed and unexposed group that may partly account for the results (46.7 vs. 40.5 years). A recent study examined the prevalence of radiation-associated posterior lens opacities among 56 interventional cardiologists and 22 controls: 52% of the former had lens opacities and only 9% of the latter [29]. Overall, relatively few cataracts have been reported among medical staff in interventional radiology, although some authors have stressed the lack of adequate monitoring [30].

In conclusion, a few studies have examined cataracts among interventional cardiologists, but the cause of the early cataracts identified has not been completely investigated. An epidemiological study in this population should provide further knowledge about the potential risk of radiation-induced cataracts in populations with exposure levels thus far considered to be low and should also improve cardiologists' awareness of the need for radiation protection. For these reasons, the Institut de Radioprotection et de Sûreté Nucléaire, with the cooperation of interventional cardiology groups of the French Society of Cardiology, has launched the O'CLOC study (Occupational Cataracts and Lens Opacities in Interventional Cardiology), designed to test the existence of an increased risk of cataracts among interventional cardiologists, compared with a control group of cardiologists not exposed to X-rays.

## Method/Design

### Study Aims

The aim of the O'CLOC study is to compare the prevalence of different stages and types of cataracts (from no opacities to severe cataracts; nuclear, cortical or posterior subcapsular) in an exposed group of interventional cardiologists with an unexposed reference group of non-interventional cardiologists, while taking into account other risk factors for cataracts, use of radiation protection tools, and exposure level.

### Population and selection

There are approximately 1700 interventional cardiologists in France: 1000 coronary interventional cardiologists (CICs) and 700 cardiologists specializing in the treatment of cardiac arrhythmias (arrhythmologists or electrophysiologists). Stratification of recruitment according to this distribution of CICs and electrophysiologists make this study the first to study both types of cardiologists exposed to X-Rays. It should therefore be as representative as possible - at least in terms of proportion - of interventional cardiology in France.

**Figure 1:**
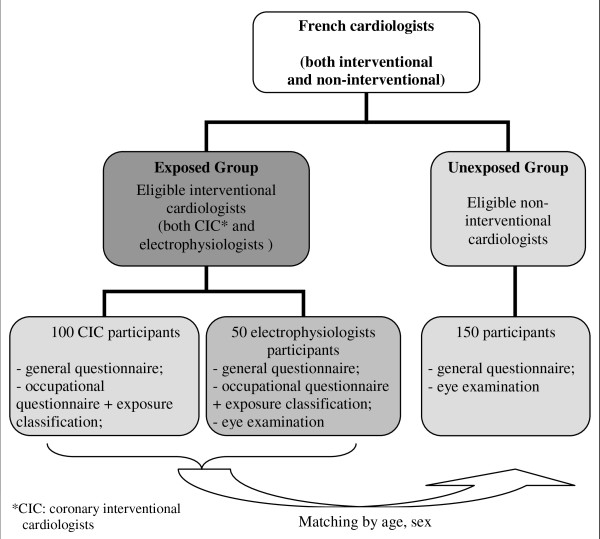
**General overview of the O'CLOC study design**.

The relative youth of the population of cardiologists has presented difficulties previously in studying cataracts in this group, specifically, the absence of data about lens opacities either in the general population for the same age class as cardiologists who could be considered as unexposed (most data concern essentially "senile cataracts"), or in an appropriate control group. That is, information about the background frequency of lens opacities in a reference population is essential. This study will compare a group of exposed individuals (interventional cardiologists chronically exposed to X-rays) to a group of unexposed but otherwise comparable individuals: cardiologists not occupationally exposed to ionizing radiation (non-interventional cardiologists). The O'CLOC design is presented in Figure 1. French centres employing CICs and arrhythmologists will be selected according to several criteria: employment of at least two interventional cardiologists; balanced distribution of the centres across France; a balanced distribution of public and private hospitals. All cardiologists in the selected centres will be contacted and invited to participate. Only cardiologists at least 40 years old will be included. This age criterion was chosen to ensure occupational exposure to IR for at least 10 years in the exposed group. Subjects with a history of personal medical radiation exposure (radiotherapy, brain scans) will be excluded from both groups. Moreover, non-interventional cardiologists with a cumulative significant history of work in interventional cardiology above one year will also be excluded. To ensure comparability between the exposed and unexposed groups, subjects will be matched by sex and age.

### Data collection

#### Questionnaires

Trained interviewers, blinded to the participants' lens opacity status, will collect all the rest of the data. Individual information will be collected about general characteristics, potential risk factors for cataracts, and history of exposure to medical radiation, both personally and occupationally. The occupational exposure data will be collected in a specific part of the questionnaire (see Table 2) that trained interviewers will use to ask the interventional cardiologists about their lifetime occupational activity. This section specifically mentions most common types of procedures: for the CICs, coronary angiography and coronary angioplasty, and for the electrophysiologists, pacemaker or intracardiac defibrillator implantation, pacemaker or intracardiac defibrillator resynchronization, radiofrequency catheter ablation except for atrial fibrillation, and radiofrequency catheter ablation of atrial fibrillation. These distinctions are justified by substantial differences in doses delivered and received by cardiologists [24].

**Table 2:** Items collected in the questionnaire

Medical information
Weight
Size
Colour of the eyes
Left or Right handed
Smoking status
Diabetes
Myopia
Corticosteroids intake
History of cancer and radiotherapy
History of treated cataract
History of eye traumatism
Congenital cataract
History of CT scans and localisation
History of significant occupational exposure to ionising radiation for more than 12 consecutive months

**Occupational history**

List of all centres and periods of activity
For each period, precise:
Coronary angiography?
Mean number of procedures per week or year
Radial or femoral access route
Coronary angioplasty?
Mean number of procedures per week or year
Radial or femoral access route
Pacemaker or intracardiac defibrillator: implantation ?
Mean number of procedures per week or year
Mean fluoroscopy time per procedure
Pacemaker or intracardiac defibrillator: resynchronisation?
Mean number of procedures per week or year
Mean fluroscopy time per procedure
Radiofrequency catheter ablation except atrial fibrillation ?
Mean number of procedures per week or year
Mean fluroscopy time per procedure
Radiofrequency catheter ablation of atrial fibrillation?
Mean number of procedures per week or year
Mean fluroscopy time per procedure
Frequency of use of radiation protection tools:
Lead apron
Lead thyroid shield
Lead eye glasses (goggles)
Other eye protection
Lead gloves
Protective mobile screen
Radiation protection cabin
Frequency of use of dosimetric badge

To supplement and verify the information collected in the occupational questionnaire, we will use data from the *SISERI System (Systéme d'Information de la Surveillance de l'Exposition aux Rayonnement Ionisants)*, an information system recording occupational dosimetry of potentially exposed French workers, centralized at the IRSN. SISERI will make it possible for us to confirm the occupationally-exposed or unexposed status of the cardiologists enrolled in the O'CLOC study. In particular, we will be able to confirm the unexposed status of eligible non-interventional cardiologists, by their absence from the database or their presence for less than 12 months. SISERI will also provide us with information on individual dosimetry monitoring (based on dosimetry badges used under the lead apron). Depending on the reliability of the doses recorded there, it may also provide us a crude estimation of doses received by cardiologists.

#### Ophthalmologic examinations

Numerous grading systems exist to detect and assess lens opacities (e.g., AREDS, Baltimore and Oxford, Merriam Focht, LOCS, etc.), and cataracts can be diagnosed according to these methods. In particular, radiation-induced cataracts have often been studied with the Merriam Focht criteria [31] and the Lens Opacities Classification System (LOCS), a normalized and internationally validated classification system used for the grading and comparison of cataract severity and type [32]. All participants in our study will undergo an ophthalmologic examination (see Table 3) that includes a slit lamp examination of the lens, to enable the diagnosis and grading of cataracts according to the LOCS III classification [32]. In practice, these examinations will be performed by volunteer ophthalmologists working in or near the cardiologists' centres. When most convenient for the cardiologist and to encourage participation, examinations could be performed by their own ophthalmologists. Both situations might result in the examiner being unmasked to the subject's exposure status, but the use of the LOCS III standardized classification should limit possible bias and ensure the reliability and repeatability of the lens opacity grading. This classification can detect various levels of lens opacities, ranging from stage 1 to stage 5 (severe). Patients with no opacities (before stage 1) are coded as "no opacity", and only they will be considered to have no cataracts. The presence of only a few lens opacities is the signature of a very early cataract or precataract status, and we consider that any stage in the LOCS III classification corresponds to a cataract, from very early (stage 1) to severe (stage 5). The LOCS classification also allows the localization of lens opacities (cortical, nuclear, posterior subcapsular) to be described and will thus permit us to analyze the potential specificity of radiation-induced cataracts.

**Table 3:** Items collected during ophthalmologic examination

For each eye:
History of treated cataract
Eyeglasses or contact lenses power (in diopters)
Measured refraction (in diopters)
Best corrected visual acuity
Intraocular pressure
LOCS III classification for lens opacities
Nuclear (Color/Opalescence)
No opacities
Stages 1 to 6
Completely opacified
Not evaluable
Cortical
No opacities
Stages 1 to 5
Completely opacified
Not evaluable
Posterior subcapsular
No opacities
Stages 1 to 5
Completely opacified
Not evaluable
Other significant pathologies (glaucoma, etc...)

#### Sample size considerations

Previous studies of interventional radiology practitioners have focused exclusively on posterior subcapsular lens opacities. Junk *et al.*, who included no unexposed group, found cataracts (advanced stages of lens opacities) in 8% of their sample. Extrapolation of the prevalences observed in the Framingham Eye Study [33], on the other hand, gave a prevalence of approximately 1.5% in the general unexposed population, Applying these figures indicates that we would need a sample size of 146 individuals in each group to have a statistical power of 80% to show a significant difference (p < 0.05). Applying the prevalence observed in other studies (eg. 37.9% in the interventional cardiologists group vs. 12% in the unexposed group in [28] or 52% to 9% in [29]) would require either 40 or 16 individuals in each group. The O'CLOC study is intended to consider a variety of different cataract types and stages, Table 1 presents other studies that have examined different exposed populations for such a variety of cataracts. In particular, with a prevalence of 23% in the exposed group [13] vs. approximately 10% in an unexposed group [33], 123 individuals in each group would be necessary. Finally, combining all this information, we estimated that the inclusion in our study of 150 subjects in each group would ensure a statistical power of 80% to test our hypothesis of a significant excess of lens opacities of different cataract types and stages in interventional cardiologists.

#### Ethical considerations

The study was approved by the local ethics committees: the CCTIRS (Advisory Committee on Information Processing in Health Research), in opinion number 09.079, and the CNIL (National Data Protection Authority) (authorization number: 909138). The Clinical Trial Registration Information is available at http://www.clinicaltrials.gov (Unique identifier NCT01061463). Participants enrolled in the study provide their written informed consent.

### Planned analysis

The non-interventional cardiologists included in the study are considered unexposed. A retrospective evaluation of the IR exposure status of the interventional cardiologists will be necessary. We will assign an exposure category level to each interventional cardiologist based on the information collected in the occupational questionnaire and from the SISERI system, as presented above. It will take into account: specialization in interventional cardiology (CICs or electophysiologists), the questionnaire information (we will consider at a minimum: duration of practice, duration of exposure and the numbers performed of each type of procedure, but other variables, such as the use of protection, handedness (left or right), equipment (film or digital), etc., will also be taken into account when possible, data from the literature (quantifying the cumulative number of examinations per physician and extrapolation from literature data about the mean dose for each type of procedure at various points in time, to estimate the total dose received), and information from the SISERI system (as presented above).

To estimate the cataract risk associated with exposure, the analysis will first compare the prevalence of all types and stages of cataracts (e.g., any type/no opacities; any type/any stage; any type/stage1; any type/stage2 cortical/no opacities; cortical/stage1; etc.) between the exposed and unexposed groups. Second, a sensitivity and specificity analysis will further analyze the outcomes to estimate the relative risk of the different types and stages of cataracts associated with interventional cardiology practices, with adjustments for matching variables (age, sex), but also for potential confounders (e.g., myopia and diabetes). Finally, the retrospective evaluation of potential exposure will be used for a qualitative study of the dose-response relation. This analysis, to the extent possible, will be based on exposure levels, will be adjusted for confounders, and will use the unexposed group as the reference group.

### Time plan for the O'CLOC study

Participant recruitment began in October 2009 and is planned to continue through January 2011. As of April 2010, 135 cardiologists (105 interventional and 30 non-interventional) have been recruited. The results should be available by 2011 and we will publish our findings, whether they are positive, negative, significant, or not significant.

## Discussion

In recent years radiation-induced cataracts have been observed in different exposed populations at lower doses than expected. Several epidemiological studies strongly suggest a non-threshold effect for these cataracts [4]. Studies in interventional medicine show that an increased risk is indeed possible. Interventional cardiology - especially electrophysiology - is a relatively new field, and no complete and well-designed epidemiological study has yet examined the possible side effects associated with these practices, such as radiation-induced cataracts. Preliminary data demonstrates the need for further investigation. Radiation cataracts tend to occur earlier than so-called senile cataracts. Cataracts remain asymptomatic for several years, so that by the time that lens opacities become optically visible and impair visual function, severe as well as irreversible damage can occur.

Recent recommendations of the ICRP determined that the data available for non-cancer diseases do not justify their inclusion in the estimation of detriment following low radiation doses [34]. The O'CLOC study is designed to provide further knowledge on the potential risk of radiation-induced cataracts, based on exposure among the population of interventional cardiologists. It will provide new evidence about the risk of radiation-induced cataracts and will help improve cardiologists' awareness of the importance of radiation protection.

## Competing interests

The authors declare that they have no competing interests.

## Authors' contributions

SJ, CS, SB, AB, OB, and MOB were responsible for identifying the research question, and contributing to drafting of the study protocol. MS, CM, PS, and DL have contributed to the development of the protocol and study design, as members of the research team. SJ, MM, and MOB were responsible for the drafting of this paper, although all authors read and approved the manuscript.

## Pre-publication history

The pre-publication history for this paper can be accessed here:

http://www.biomedcentral.com/1471-2458/10/537/prepub

## References

[B1] ICPR The 1991 Recommendations of International Commission on Radiological Protection ICPR Publication 60 1991 Oxford: Pergamon Press

[B2] NCPR Limitation of exposure to ionizing radiation Report 116 1993 Bethesda, MD: National Council on Radiation Protection and Measurements

[B3] Ainsbury EA Bouffler SD Dorr W Graw J Muirhead CR Edwards AA Cooper J Radiation cataractogenesis: a review of recent studies Radiation research 2009 172 1 1 9 10.1667/RR1688.1 19580502

[B4] Kleiman N Euratom, Radiation protection 145 - New insights in radiation risk and basic safety standards 2007 81 95 (Radiation cataract)

[B5] Otake M Schull WJ Radiation-related posterior lenticular opacities in Hiroshima and Nagasaki atomic bomb survivors based on the DS86 dosimetry system Radiation research 1990 121 1 3 13 10.2307/3577557 2300666

[B6] Minamoto A Taniguchi H Yoshitani N Mukai S Yokoyama T Kumagami T Tsuda Y Mishima HK Amemiya T Nakashima E Cataract in atomic bomb survivors International journal of radiation biology 2004 80 5 339 345 10.1080/09553000410001680332 15223766

[B7] Nakashima E Neriishi K Minamoto A A reanalysis of atomic-bomb cataract data, 2000-2002: a threshold analysis Health physics 2006 90 2 154 160 10.1097/01.HP.0000175442.03596.63 16404173

[B8] Day R Gorin MB Eller AW Prevalence of lens changes in Ukrainian children residing around Chernobyl Health physics 1995 68 5 632 642 10.1097/00004032-199505000-00002 7730059

[B9] Chen WL Hwang JS Hu TH Chen MS Chang WP Lenticular opacities in populations exposed to chronic low-dose-rate gamma radiation from radiocontaminated buildings in Taiwan Radiation research 2001 156 1 71 77 10.1667/0033-7587(2001)156[0071:LOIPET]2.0.CO;2 11418075

[B10] Klein BE Klein R Linton KL Franke T Diagnostic x-ray exposure and lens opacities: the Beaver Dam Eye Study American journal of public health 1993 83 4 588 590 10.2105/AJPH.83.4.588 8460743 PMC1694473

[B11] Hall P Granath F Lundell M Olsson K Holm LE Lenticular opacities in individuals exposed to ionizing radiation in infancy Radiation research 1999 152 2 190 195 10.2307/3580093 10409329

[B12] Wilde G Sjostrand J A clinical study of radiation cataract formation in adult life following gamma irradiation of the lens in early childhood The British journal of ophthalmology 1997 81 4 261 266 10.1136/bjo.81.4.261 9215051 PMC1722161

[B13] Worgul BV Kundiyev YI Sergiyenko NM Chumak VV Vitte PM Medvedovsky C Bakhanova EV Junk AK Kyrychenko OY Musijachenko NV Cataracts among Chernobyl clean-up workers: implications regarding permissible eye exposures Radiation research 2007 167 2 233 243 10.1667/RR0298.1 17390731

[B14] Cucinotta FA Manuel FK Jones J Iszard G Murrey J Djojonegro B Wear M Space radiation and cataracts in astronauts Radiation research 2001 156 5 Pt 1 460 466 10.1667/0033-7587(2001)156[0460:SRACIA]2.0.CO;2 11604058

[B15] Rastegar N Eckart P Mertz M Radiation-induced cataract in astronauts and cosmonauts Graefe's archive for clinical and experimental ophthalmology = Albrecht von Graefes Archiv fur klinische und experimentelle Ophthalmologie 2002 240 7 543 547 12136284 10.1007/s00417-002-0489-4

[B16] Chylack LT Jr Peterson LE Feiveson AH Wear ML Manuel FK Tung WH Hardy DS Marak LJ Cucinotta FA NASA study of cataract in astronauts (NASCA). Report 1: Cross-sectional study of the relationship of exposure to space radiation and risk of lens opacity Radiation research 2009 172 1 10 20 10.1667/RR1580.1 19580503

[B17] Rafnsson V Olafsdottir E Hrafnkelsson J Sasaki H Arnarsson A Jonasson F Cosmic radiation increases the risk of nuclear cataract in airline pilots: a population-based case-control study Archives of ophthalmology 2005 123 8 1102 1105 10.1001/archopht.123.8.1102 16087845

[B18] Chodick G Bekiroglu N Hauptmann M Alexander BH Freedman DM Doody MM Cheung LC Simon SL Weinstock RM Bouville A Risk of cataract after exposure to low doses of ionizing radiation: a 20-year prospective cohort study among US radiologic technologists American journal of epidemiology 2008 168 6 620 631 10.1093/aje/kwn171 18664497 PMC2727195

[B19] Scanff P Donadieu J Pirard P Aubert B Population exposure to ionizing radiation from medical examinations in France The British journal of radiology 2008 81 963 204 213 10.1259/bjr/24344062 18270294

[B20] Bhargavan M Trends in the utilization of medical procedures that use ionizing radiation Health physics 2008 95 5 612 627 10.1097/01.HP.0000327659.42618.c1 18849695

[B21] Togni M Balmer F Pfiffner D Maier W Zeiher AM Meier B Percutaneous coronary interventions in Europe 1992-2001 European heart journal 2004 25 14 1208 1213 10.1016/j.ehj.2004.04.024 15246638

[B22] Delichas M Psarrakos K Molyvda-Athanassopoulou E Giannoglou G Sioundas A Hatziioannou K Papanastassiou E Radiation exposure to cardiologists performing interventional cardiology procedures European journal of radiology 2003 48 3 268 273 10.1016/S0720-048X(03)00007-X 14652145

[B23] Vano E Radiation exposure to cardiologists: how it could be reduced Heart (British Cardiac Society) 2003 89 10 1123 1124 12975391 10.1136/heart.89.10.1123 PMC1767905

[B24] Kim KP Miller DL Balter S Kleinerman RA Linet MS Kwon D Simon SL Occupational radiation doses to operators performing cardiac catheterization procedures Health physics 2008 94 3 211 227 10.1097/01.HP.0000290614.76386.35 18301095

[B25] Padovani R Vano E Trianni A Bokou C Bosmans H Bor D Jankowski J Torbica P Kepler K Dowling A Reference levels at European level for cardiac interventional procedures Radiation protection dosimetry 2008 129 1-3 104 107 10.1093/rpd/ncn039 18310612

[B26] Vano E Faulkner K ICRP special radiation protection issues in interventional radiology, digital and cardiac imaging Radiation protection dosimetry 2005 117 1-3 13 17 10.1093/rpd/nci702 16461540

[B27] Junk A Haskal Z Worgul B Cataract in interventional radiology - An occupational hazard ? Invest Ophthalmol Vis Sci 2004;45: E-Abstract 388 2004 45 E Abstract 388

[B28] Duran D Duran G Ramirez R Vano E Kleinman N Echeverri D Gomez G Cabrera M Cataracts in interventional cardiology personnel. Retrospective evaluation study of lens injuries and dose (RELID Study) European heart journal 2009 30 Abstract supplement 872

[B29] Ciraj-Bjelac O Rehani MM Sim KH Liew HB Vano E Kleiman NJ Risk for radiation induced cataract for staff in interventional cardiology: Is there reason for concern? Catheter Cardiovasc Interv 2010 20549683 10.1002/ccd.22670

[B30] Vano E Gonzalez L Beneytez F Moreno F Lens injuries induced by occupational exposure in non-optimized interventional radiology laboratories The British journal of radiology 1998 71 847 728 733 9771383 10.1259/bjr.71.847.9771383

[B31] Merriam GR Jr Szechter A Focht EF The effects of ionizing radiations on the eye Front Radiat Ther Oncol 1972 5 346 385

[B32] Chylack LT Jr Wolfe JK Singer DM Leske MC Bullimore MA Bailey IL Friend J McCarthy D Wu SY The Lens Opacities Classification System III. The Longitudinal Study of Cataract Study Group Archives of ophthalmology 1993 111 6 831 836 8512486 10.1001/archopht.1993.01090060119035

[B33] Leibowitz HM Krueger DE Maunder LR Milton RC Kini MM Kahn HA Nickerson RJ Pool J Colton TL Ganley JP The Framingham Eye Study monograph: An ophthalmological and epidemiological study of cataract, glaucoma, diabetic retinopathy, macular degeneration, and visual acuity in a general population of 2631 adults, 1973-1975 Survey of ophthalmology 1980 24 Suppl 335 610 7444756

[B34] ICPR The 2007 Recommendations of International Commission on Radiological Protection ICPR Publication 103 2007 Oxford: Elsevier

